# Postpartum urinary retention: Evaluation of risk factors

**DOI:** 10.4274/tjod.43931

**Published:** 2018-06-21

**Authors:** Mesut Polat, Mehmet Baki Şentürk, Çiğdem Pulatoğlu, Ozan Doğan, Çetin Kılıççı, Mehmet Şükrü Budak

**Affiliations:** 1 İstanbul Medeniyet University Faculty of Medicine, Departments of Obstetrics and Gynecology, İstanbul, Turkey; 2 Bayburt State Hospital, Clinic of Obstetrics and Gynecology, Bayburt, Turkey; 3 University of Health Sciences, Şişli Hamidiye Etfal Training and Research Hospital, Clinic of Obstetrics and Gynecology, İstanbul, Turkey; 4 İstanbul Zeynep Kamil Women and Children’s Diseases Training and Research Hospital, Clinic of Obstetrics and Gynecology, İstanbul, Turkey; 5 University of Health Sciences, Diyarbakır Gazi Yaşargil Training and Research Hospital, Clinic of Obstetrics and Gynecology, Diyarbakır Turkey

**Keywords:** Postpartum urinary retention, postpartum bladder dysfunction, risk factors

## Abstract

**Objective:**

Postpartum urinary retention means the absence of spontaneous micturition more than 6 hours after birth or when residual volume after urination is less than 150 cc. If neglected, postpartum urinary retention may result in bladder denervation and detrusor muscle weakness requiring intermittent catheterization or permanent micturition dysfunction. Our goal was to identify the possible risk factors for postpartum urinary retention.

**Materials and Methods:**

Five hundred sixty female subjects were included in this retrospective study. All data obtained including variables such as age, parity, body mass index, duration of labor, prepartum bladder catheterization were compared between female subjects with and without postpartum urinary retention.

**Results:**

Among the 560 patients recruited to our study, 124 (22.1%) had postpartum urinary retention. Third stage duration, time from birth to the first void, and number of peripartum micturitions were found to be potential risk factors for postpartum urinary retention. Different than other studies, our study revealed a correlation between peripartum catheterization and postpartum urinary retention. There were no statistically significant differences between patients with and without postpartum urinary retention in terms of other variables.

**Conclusion:**

In this study, a correlation between peripartum catheterization and postpartum urinary retention was found. There are studies that reported the possible risk factors related to the occurrence of postpartum urinary retention. More studies should be conducted to investigate long-term results with larger populations.

**PRECIS:** To identify the possible risk factors for postpartum urinary retention.

## Introduction

Postpartum voiding dysfunction is defined as having difficulty in complete micturition leading to urine retention or an inability to urinate spontaneously after giving birth^(1)^. The exact incidence is not clear because of undiagnosed asymptomatic cases, but according to the literature, the estimated incidence of postpartum urinary retention (PPUR) has a wide range between 0.05% and 37% depending on the definitions used^(2)^. Overt PPUR is defined as the failure in spontaneous voiding within six hours of vaginal birth, whereas covert PPUR refers to a bladder volume of ≥150 mL remaining after spontaneous urination^(3)^. Although PPUR has an uncertain pathophysiology, there are several hypotheses on the cause of PPUR. Vaginal delivery can be traumatic for pelvic floor muscles and innervations, which can cause reduced bladder sensitivity. Also, peri-urethral and vulvar edema due to vaginal delivery may result in obstruction. Many prognostic factors have been identified such as mode and duration of labor, the presence of perineal trauma, the method of anesthesia or analgesia, the body mass index (BMI) of the patient, and the birth weight of the baby. The long-term effects of PPUR have not yet been identified, but many complications of persistent urinary retention due to non-delivery causes have been described. If neglected, urinary retention may result in denervation of the bladder, detrusor muscle weakness and failure, anuria, hydronephrosis, and even kidney failure caused by renal obstruction^(4,5)^. Whether specific treatment is unnecessary or requires intermittent catheterization may be self-limiting^(6)^. Screening for PPUR is not part of standard postpartum care; therefore, identifying the risk factors and early diagnosis are as important as appropriate management to prevent the potential damage of enduring retention. Our aim was to describe the possible risk factors for postpartum bladder dysfunction.

## Materials and Method

We conducted a retrospective study of 560 women who have birth from January 2014 to September 2016 at a single tertiary center. The study was approved by the Ethics Committee of University of Health Sciences, Diyarbakır Gazi Yaşargil Training and Research Hospital in Turkey (approval number: 2018-30). Approval of the local ethics committee was provided for the study and the study protocol adhered to the principles of the Declaration of Helsinki. Patients who were older than 18 years and delivered via vaginal birth were included in the study. Patients who had urinary tract disease, overactive bladder, pelvic organ prolapsus or previous bladder surgery were excluded. Demographic data such as age, parity, BMI, and obstetric variables such as the use of augmentation with oxytocin or Propess^®^, duration of active phase of labor, presence of episiotomy, degree of laceration, birth weight of the baby, number of voids during delivery, and peripartum bladder catheterization were documented and analyzed. The patients were considered as having PPUR when there was no micturition within 6 hours of delivery. For patients who were suspected as having PPUR immediately after the first micturition, residual urine volume was measured using transabdominal ultrasonography (USG) (Logiq Pro 200, 3.5 MHz convex transducer probe) and these data were documented from the patients’ medical files. Estimated post voiding bladder volume was calculated using the formula, D1xD2xD3x0.9 (D1: the widest diameter in the transverse scan, D2: anteroposterior diameter in the longitudinal scan, D3: cephalocaudal diameter in the longitudinal scan)^(7)^. Assessment of urinary retention during the peripartum period is difficult and there is no consensus on the amount of bladder volume that is considered as urinary retention. Generally, a normal post-micturition residual volume is accepted as 50-200 mL in women^(8)^. Bladder volume can be measured by catheterization with considerable risk of urinary infection. Transabdominal USG is a non-invasive method for the assessment of urinary retention but its accuracy may be limited especially during prepartum because of the fetus^(9)^. However, in this study, USG was used without this limitation because PPUR was assessed.

We defined PPUR as a failure in spontaneous voiding within six hours of birth or post void bladder volume >150 mL in accordance with the literature, as verified using USG^(10)^. All obtained data were compared between women with and without PPUR.

### Statistical Analysis

The statistical analyses were performed using the Statistical Package for the Social Sciences software, Version 17.0. Categorical data were compared using the chi-square test, Fisher’s exact test, and Student’s t-test for normal distribution, or the Mann-Whitney U test for those with non-normal distribution. P<0.05 was considered statistically significant. Significant variables were analyzed by bivariate and multivariate logistic regression analyses to determine which factors were independently associated with PPUR. Odds ratio and corresponding 95% confidence intervals are reported.

## Results

Among the 560 patients recruited to our study, the mean ages of patients with and without PPUR were 27.90±6.69 years and 27.19±6.83 years, respectively. The incidence of PPUR was 22.1% (n=124). There were no statistically significant differences between patients with and without PPUR regarding age, BMI, gravida, parity, induction with oxytocin or Propess^®^, use of vacuum or forceps, duration of the second and third stage, fundal pressure, peripartum catheterization, number of micturitions, post-micturition residual volume, birth weight of the baby, presence of episiotomy or deep perineal laceration, and duration of the active phase. Table 1 describes the demographic data of women and labor characteristics with and without PPUR. When compared with patients without PPUR, the duration of the third stage was longer in women with PPUR (13.57±10.31 minutes vs. 17.02±12.93 minutes). The number of peripartum micturitions was significantly higher in women without PPUR than patients with PPUR (p<0.001). The analyses showed that the time from birth to the first void was significantly longer in women with PPUR (661.02±1372.77 minutes vs. 212.57±80.00 minutes). No significant differences were found in terms of other variables. When the factors affecting the development of PPUR were analyzed, it was shown that a 10 minute increase in the second stage of labor led to a 6% increase in the risk of PPUR; a 1 minute increase in time from birth to the first void increased PPUR risk by 4%. The absence of prepartum bladder catheterization was related to 2.2-fold increased risk of PPUR development. Given the effect of the number of peripartum voids on PPUR, an increase of one in the number of voids reduced the risk by 24.1%. No statistically significant effect of other variables on the occurrence of PPUR was found (Table 2).

## Discussion

We found that PPUR was relatively common with an incidence of 22.1%. The reported incidence in the literature varies between 0.05-37%^(2,11)^. These differences may be due to different study designs and under diagnosis of covert retention. However, many of the cases remain undiagnosed and most patients with PPUR have no symptoms. The average maternal age ranges between 25 and 28 years in the literature^(12)^. In our study, the mean age was also within these ranges for patients with and without PPUR (27.90±6.69 years, 27.19±6.83 years, respectively). The pathophysiology of PPUR is still unclear. Some physiologic, neurologic, and mechanical causes may be responsible for PPUR development. The detrusor muscle can be inhibited by the effect of increased progesterone level, thereby leading to urinary retention. Vaginal delivery can be traumatic for pelvic floor muscles and innervations, which can result in hypotonicity or reduced bladder sensitivity. Also, peri-urethral and vulvar edema due to vaginal delivery may result in obstruction^(13,14)^. In the literature, many risk factors have been identified for the occurrence of PPUR. The development of PPUR may be caused by prolonged stages of delivery. We found that prolonged second and third stage of labor increased the risk of PPUR, but the duration of the active phase was not associated with PPUR. A possible mechanism is applied mechanical strength, which contributes to pelvic nerve damage leading to neurologic impairment of the bladder. In some studies, the association of prolonged labor with PPUR has also been reported^(6,12)^. Similarly, in a study conducted by Kekre et al.,^(3)^ it was reported that PPUR rates were higher in patients with a prolonged second stage of labor. Salemnic et al.^(6)^ claimed that there was a relationship between PPUR and longer second stage of birth and mediolateral episiotomy, yet this study was performed on 200 women who delivered vaginally and only once. In a larger study conducted by Yip et al.,^(12)^ only women with vaginal deliveries (n=691) were studied and it was found that a duration of delivery more than 800 minutes was significantly correlated with a rise in PPUR. In the present study, we found no relationship between parity and PPUR development. Nulliparity is usually perceived as a risk factor^(1,14)^. Nulliparous women are thought to be exposed to pelvic floor tenderness and pudendal nerve damage in the course of vaginal birth. The risk of developing PPUR was higher in primigravida than multigravida women in a study conducted by Liang et al.^(15)^. In contrast to our findings, Pifarotti et al.^(16)^ reported that fundal pressure was another risk factor for PPUR. Unlike our study, many studies reported that perineal damage had an impact on the development of PPUR^(17,18)^. A study by Musselwhite et al.^(17)^ revealed that second- and third-degree perineal tears, which might result in reflex urethral spasm, had a relationship with PPUR. However, in the same study, episiotomy was found to have no impact on PPUR^(17)^. The risk of PPUR was increased by perineal tears including sphincter rupture in a study conducted by Glavind and Bjork^(18)^. In contrast, Yip et al.^(12)^ reported that perineal trauma had no effect on the incidence of PPUR.

Contrary to other authors^(3,19)^, we detected no association between vaginal operative birth and PPUR. Vaginal operative delivery can be confused with other factors such as longer delivery, epidural analgesia, parity and episiotomy, and causes debates regarding whether it is an independent predictor^(1)^. Several studies, as well as ours, have shown that fetal birth weight does not increase the risk of PPUR^(8,20)^. Conversely, it has been reported that PUR was also due to infant birthweight lower than 3800 g^(14)^. In our study, we did not document the follow-up of the patients. In a study by Carley et al.,^(11)^ 11.332 women were evaluated following vaginal delivery and it was found that 45% of symptomatic PPUR resolved within 48 hours and 25% of women had persistence for more than 72 hours. Various short-term complications of PPUR have been identified, but it is not certain whether PPUR is related to any long-term morbidity. Women with PPUR were not significantly different from those without PPUR in the study by Yip et al.,^(21) ^which was a four-year follow-up about women with PPUR, using outcome variables such as urinary stress incontinence, fecal incontinence, frequency, nocturia, urgency, urge incontinence, and coital incontinence. In a larger study, it was reported that 0.05% of PPUR had persisted related to long-term bladder dysfunction^(19)^. There is no consensus on the significance of PPUR^(22)^. Furthermore, guidelines do not recommend routine bladder scanning for the diagnosis of PPUR, due to the size of the uterus after delivery, and the accuracy of USG measurements of residual volume is debatable^(23,24)^. More research is needed to confirm the benefits and cost-effectiveness of post-routine bladder screening. The present study demonstrates that the PPUR development rate is lower in patients who had peripartum bladder catheterization. There are many studies in the literature reporting the possible risk factors related to the occurrence of PPUR, but most do not mention peripartum bladder catheterization, which makes it difficult to compare them with results in terms of the effect of catheterization on the development of PPUR. To the best of our knowledge, this is the first study to define the relationship between peripartum catheterization and the development of PPUR.

### Study Limitations

One of the limitations of the study is it’s retrospective and the small number of cases. Another point is that it did not include volumes of voiding or oral intake of the women.

## Conclusion

In conclusion, PPUR is a relatively common problem that can lead to irreversible damage to the bladder. Further prospective studies with more participants are needed to support the findings and define long-term implications of PPUR for detecting, avoiding, and managing postpartum voiding dysfunction.

## Figures and Tables

**Table 1:**
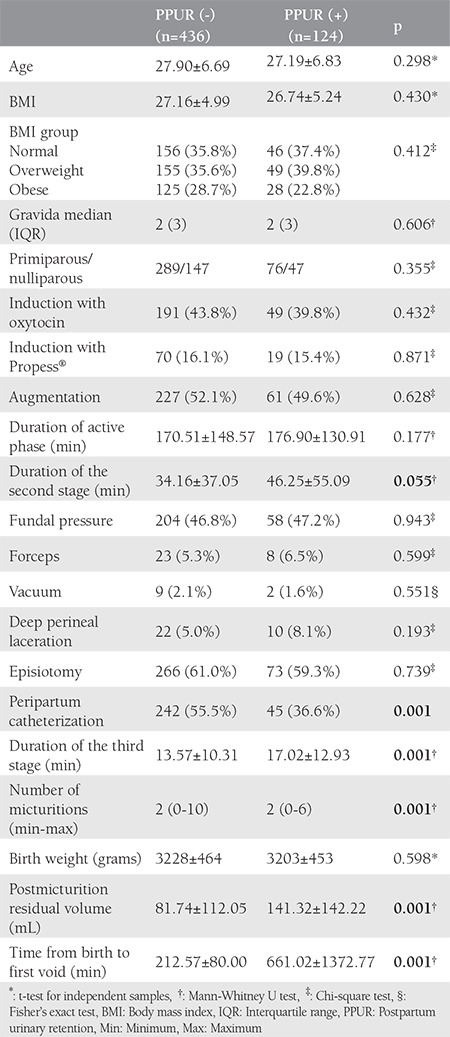
Characteristics of patients and labor with or without postpartum urinary retention

**Table 2:**
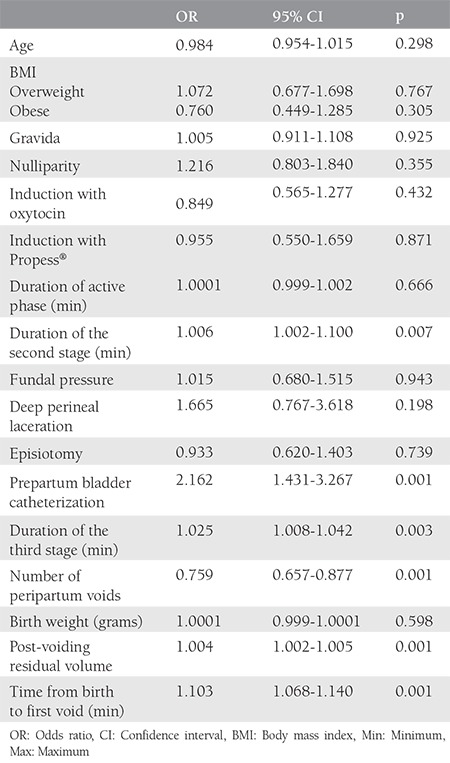
Factors affecting the development of postpartum urinary retention (bivariate logistic regression)

## References

[1] Mulder FE, Schoffelmeer MA, Hakvoort RA, Limpens J, Mol BW, van der Post JA, et al (2012). Risk factors for postpartum urinary retention: a systematic review and meta-analysis. BJOG.

[2] Buchanan J, Beckmann M (2014). Postpartum voiding dysfunction: identifying the risk factors. Aust N Z J Obstet Gynaecol.

[3] Kekre AN, Vijayanand S, Dasgupta R, Kekre N (2011). Postpartum urinary retention after vaginal delivery. Int J Gynaecol Obstet.

[4] Mustonen S, Ala-Houhala IO, Tammela TL (2001). Long-term renal dysfunction in patients with acute urinary retention. Scand J Urol Nephrol.

[5] Zaki MM, Pandit M, Jackson S (2004). National survey for intrapartum and postpartum bladder care: assessing the need for guidelines. BJOG.

[6] Salemnic Y, Gold R, Toov JH, Jaffa A, Gordon D, Lessing J, et al (2012). Prevalence, obstetric risk factors and natural history of asymptomatic postpartum urinary retention after first vaginal delivery - a prospective study of 200 primipara women. J Urol.

[7] Poston GJ, Joseph AE, Riddle PR (1983). The accuracy of ultrasound in the measurement of changes in bladder volume. Br J Urol.

[8] MacLean AB, Cardozo L (2002). Incontinence in Women - Study Group Statement. London: Royal College of Obstetricians and Gynaecologists Press.

[9] Gyampoh B, Crouch N, O’Brien P, O’Sullivan C, Cutner A (2004). Intrapartum ultrasound estimation of total bladder volume. BJOG.

[10] World Health Organization (2010.). World Health Organization Technical Consultation on Postpartum and Postnatal Care. Geneva: World Health Organization.

[11] Carley ME, Carley JM, Vasder G, Lesnick TG, Webb MJ, Ramin KD (2002). Factors that are associated with clinically overt postpartum urinary retention after vaginal delivery. Am J Obstet Gynecol.

[12] Yip SK, Brieger G, Hin LY, Chung T (1997). Urinary retention in the post-partum period. The relationship between obstetric factors and the post-partum post-void residual bladder volume. Acta Obstet Gynecol Scand.

[13] Teo R, Punter J, Abrams K, Mayne C, Tincello D (2007). Clinically overt postpartum urinary retention after vaginal delivery: a retrospective case-control study. Int Urogynecol J Pelvic Floor Dysfunct.

[14] Lim JL (2010). Post-partum voiding dysfunction and urinary retention. Aust N Z J Obstet Gynaecol.

[15] Liang CC, Chang SD, Chang YL, Chen SH, Chueh HY, Cheng PJ (2007). Postpartum urinary retention after cesarean delivery. Int J Gynaecol Obstet.

[16] Pifarotti P, Gargasole C, Folcini C, Gattei U, Nieddu E, Sofi G, et al (2014). Acute post-partum urinary retention: analysis of risk factors, a case-control study. Arch Gynecol Obstet.

[17] Musselwhite KL, Faris P, Moore K, Berci D, King KM (2007). Use of epidural anesthesia and the risk of acute postpartum urinary retention. Am J Obstet Gynecol.

[18] Glavind K, Bjork J (2003). Incidence and treatment of urinary retention postpartum. Int Urogynecol J Pelvic Floor Dysfunct.

[19] Groutz A, Hadi E, Wolf Y, Maslovitz S, Gold R, Lessing JB, et al (2004). Early postpartum voiding dysfunction: incidence and correlation with obstetric parameters. J Reprod Med.

[20] Rizvi RM, Khan ZS, Khan Z (2005). Diagnosis and management of postpartum urinary retention. Int J Gynaecol Obstet.

[21] Yip SK, Sahota D, Chang AM, Chung TK (2002). Four-year follow-up of women who were diagnosed to have postpartum urinary retention. Am J Obstet Gynecol.

[22] Shah JPR, Dasgupta P (2000). Voiding difficulties and retention. Clinical Urogynaecology, 2nd edn. London: Churchill Livingston.

[23] Routine Postnatal Care of Women and their Babies (2006.). National Institute for Health and Clinical Excellence Guideline 37. London;.

[24] World Health Organization (2010.). World Health Organization Technical Consultation on Postpartum and Postnatal Care. Geneva: World Health Organization.

